# School’s Out: Seasonal Variation in the Movement Patterns of School Children

**DOI:** 10.1371/journal.pone.0128070

**Published:** 2015-06-01

**Authors:** Adam J. Kucharski, Andrew J. K. Conlan, Ken T. D. Eames

**Affiliations:** 1 Centre for the Mathematical Modelling of Infectious Diseases, London School of Hygiene & Tropical Medicine, London, UK; 2 Fogarty International Center, National Institutes of Health, Bethesda, USA; 3 Disease Dynamics Unit, Department of Veterinary Medicine, University of Cambridge, Cambridge, UK; Centre de Physique Théorique, FRANCE

## Abstract

School children are core groups in the transmission of many common infectious diseases, and are likely to play a key role in the spatial dispersal of disease across multiple scales. However, there is currently little detailed information about the spatial movements of this epidemiologically important age group. To address this knowledge gap, we collaborated with eight secondary schools to conduct a survey of movement patterns of school pupils in primary and secondary schools in the United Kingdom. We found evidence of a significant change in behaviour between term time and holidays, with term time weekdays characterised by predominately local movements, and holidays seeing much broader variation in travel patterns. Studies that use mathematical models to examine epidemic transmission and control often use adult commuting data as a proxy for population movements. We show that while these data share some features with the movement patterns reported by school children, there are some crucial differences between the movements of children and adult commuters during both term-time and holidays.

## Introduction

The social mixing behaviour of school-aged children is believed to be an important driving factor of infectious disease spread. Levels of susceptibility and social mixing are likely to be higher amongst school children than any other age group, and the relatively high levels of incidence of many common infections in children identifies them as an important epidemiological group [1–5].

Significant efforts have been made in recent years to quantify human social mixing patterns with a view to improving our understanding of the transmission dynamics of infection [6–11]. There has been a particular focus on quantifying the amount of interaction between different age groups. Studies have consistently shown that school-aged children make large numbers of social contacts, predominantly with other school-aged children [8, 12]. Recent work has quantified the extent to which mixing patterns change during school holiday periods; as might be expected, children make fewer contacts during school holidays, with the greatest reduction being in the number of contacts with other children [13, 14].

These social mixing studies have provided the essential information necessary to interpret age-stratified incidence patterns of common infectious diseases. Likewise, information on the movement of individuals is essential for understanding, and predicting, the spatial spread of infection. Mathematical models have been developed to predict the spatial dispersal of infection based upon human movement data [15–19]. Currently, available movement data are largely restricted to commuting information [20–22], long distance travel (e.g. air-travel [23, 24]) and a variety of proxy measures, from banknotes [25] to mobile phones [26]. While all of these sources are likely to be useful, none provide much information about the movements of school-aged children, the key drivers of transmission for many common infectious diseases. Some countries, such as Switzerland, do conduct more detailed diary-based studies of travel patterns [27], which have been used to inform mathematical models [28, 29]. However, such data is not yet available for the United Kingdom.

Here, we present the results of a study of the spatial movement patterns of school children, carried out in sixteen schools across the UK. Movements were measured both during school term times and school holidays, allowing a picture to be built up of the seasonal changes in spatial mixing behaviour of children.

## Materials and Methods

We worked with groups of schools during the 2011–12 and 2012–13 academic years to devise and carry out a project to measure movement patterns of school children during term time and school holidays. The methodology was based on an earlier project in which secondary school pupils quantified the social networks present in their local primary schools [30].

During each academic year we worked alongside 4 secondary schools to design a questionnaire, collect, and analyse data. Interaction was mainly through video-conferences, which allowed the school students to share ideas with each other as well as with the researchers, and visits from the researchers to the schools. The project was conceived partly as public engagement, partly as outreach, and partly as research [31]; one of the main aims of the project was to give support to students in developing their research questions and methods.

During the development phase of the study, students worked to devise a survey that would quantify the distance travelled by school pupils, both in term time and holidays. The final questionnaire was agreed upon by all schools, and a consistent version used throughout the project. It was intended to use the questionnaire in both secondary and primary schools, so it was designed to be as simple to complete as possible. Pupils and schools planned the details of their engagement with participants to best suit their local circumstances.

The final survey asked participants to report, for each day over a two week period (including one week of term time and one week of school holidays), the furthest they were from home that day. The question was posed both as a set of distance options (at home all day; < 1 mile; 1–5 miles; 5–30 miles; 30–100 miles; > 100 miles) and by asking for the location (either as a place name, street name, or postcode). Where places were reported, these were converted into postcodes (using the first half of the postcode only) where possible.

Questionnaires were distributed with explanation at start of the fortnight, and could be filled in by participants or by their parents on their behalf, or by a combination of the two; it was left for individual families to decide on the approach that best suited them. To reduce recall errors, participants were encouraged to complete the survey daily during the study period. In the first year the study period included a week of the Easter holiday; in the second year the study period included the week-long Spring half term holiday. This change was motivated both by the need to fit the project in around the school timetable, and as a result of suggestions by participating schools that it would lead to an improved return rate.

For analysis purposes, we categorised days as ‘term time weekdays’, ‘holiday weekdays’ or ‘weekends’ and compared reported movements during these periods by calculating the proportion of reported trips that fell into each distance category. We calculated bootstrapped confidence intervals for these proportions by repeatedly resampling the data with replacement to obtain multiple alternate datasets of the same size. To compare school patterns with adult movements, we also measured the commuting distances reported in the 2001 census in the 17 UK electoral wards that covered our study locations [32]. Across these wards, in the 2001 census 12,792 adults reported commuting movements [33]. Distances were measured from the centroid of the home ward to the centroid of the work ward, and assigned to the same set of categories as used in the school questionnaire, except that we did not include a category for ‘at home’ because these data were not available.

In addition to providing estimates for the maximum distance travelled, participants were also encouraged to report the (full or partial) postcode of the furthest location from home to which they travelled. Postcodes were then geo-referenced to UK national grid co-ordinates using the Office of National Statistics Postcode Directory (ONSPD). These data allowed the creation of maps demonstrating the approximate location of movements. Locations with an incomplete postcode were assigned a unique geo-location using the centroid of all partially matching postcodes. Mapped locations were jittered uniformly within 2.5km squares to avoid over plotting and to preserve anonymity. Maps were created using digital boundaries and databases provided by the Office of National Statistics through the Open Geography portal.

Participation in this opt-in study was voluntary, and informed written consent was obtained from parents/guardians on behalf of the children taking part in the study. All analysis was carried out on anonymised data. The study was approved by the ethics committee of the London School of Hygiene & Tropical Medicine.

## Results

During the 2011–12 and 2012–13 academic years, the travel movement questionnaire was given to students in 16 schools in urban and rural locations around the UK. 825 questionnaires were returned with sufficient information (age and/or year group of participant) to be included in the final database (S1 dataset). 208 were returned in the first year, and 617 in the second year. Details of the number of questionnaires given out by each school group were not available in every case; the return rate was approximately 10% in the first year, and in the second year, where we have this information, the return rate was approximately 42% (520 out of 1226 surveys returned). Returned surveys were reasonably complete; 2.6% of distance reports (2.9% in year 1 and 2.5% in year 2) and 8.6% of postcode reports were missing (6.4% in year 1, 9.3% in year 2).

**Fig 1:**
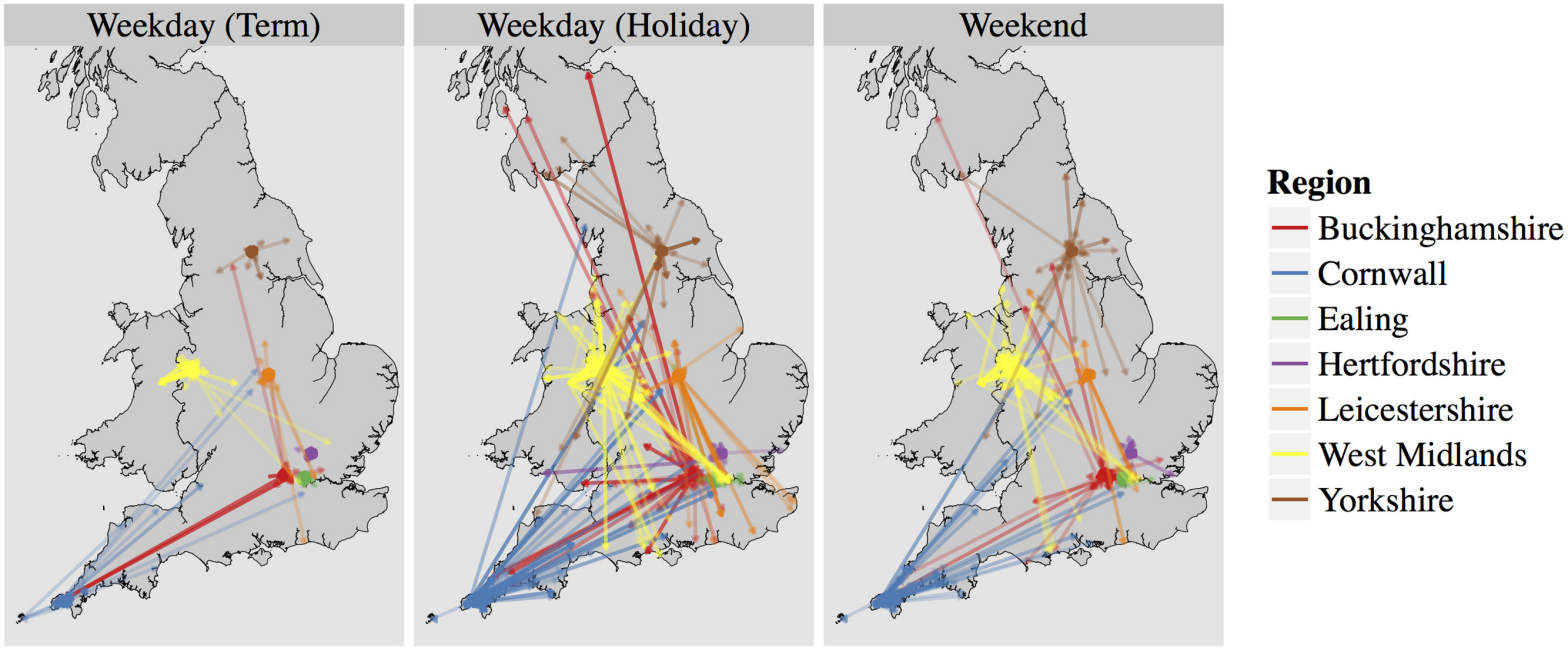
Furthest distance reported by participants during term weekdays (2,653 movements), holiday weekdays (3,086 movements) and weekends (2,540 movements). Arrows indicate direction of travel; colours show location of different schools in the survey, grouped by district. Point locations are jittered uniformly within 2.5km grids to preserve anonymity and reduce over plotting.

The geographic distribution of reported movements is shown in Fig 1. We found a distinct difference in movement patterns between term time and holidays. During term-time weekdays, most trips cluster around the home location, whereas travel patterns span a markedly wider area during holidays and weekends. The long distance trips from Buckinghamshire to Cornwall in term-time were the result of a class trip.

**Fig 2:**
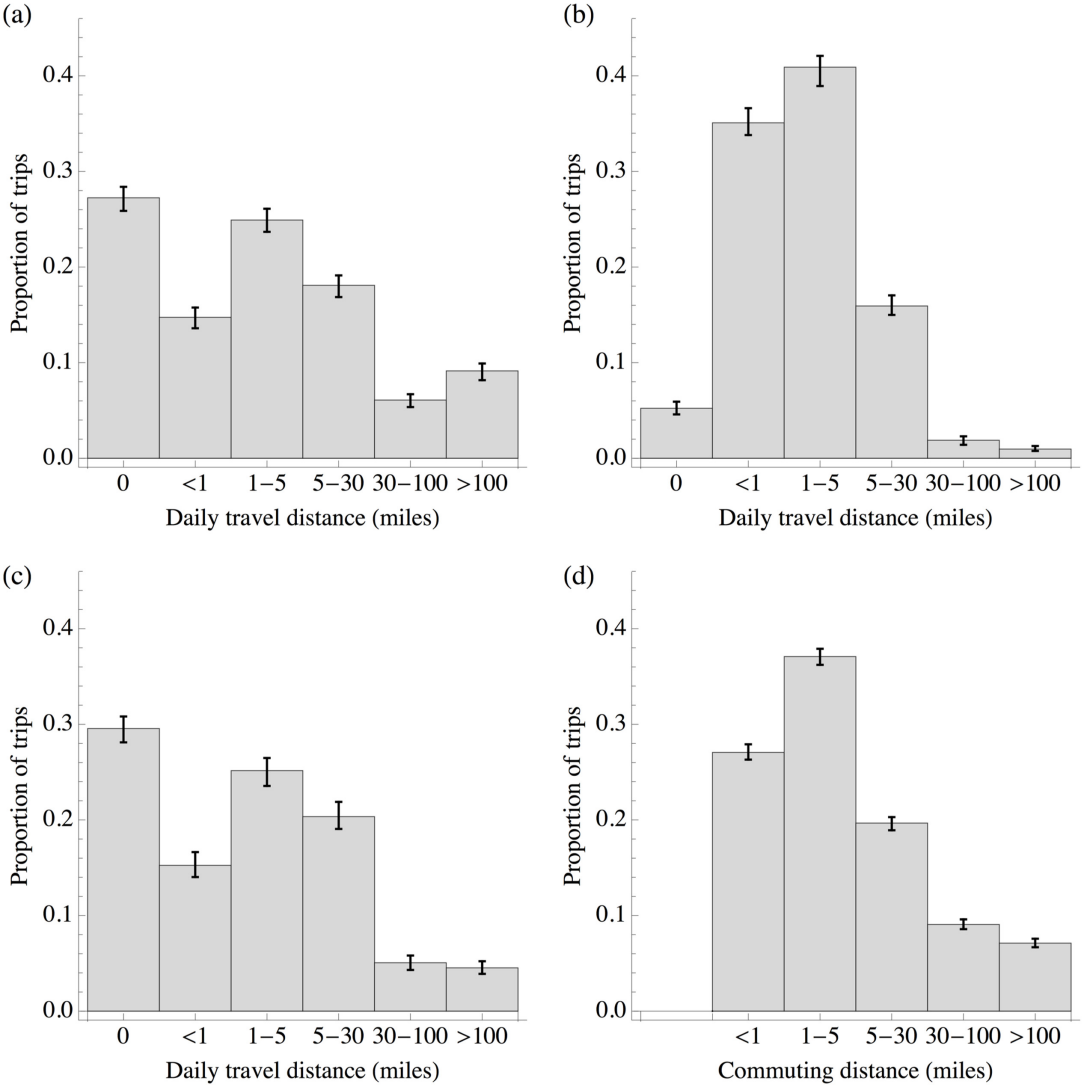
Comparison of reported movement distances. (A) Travel patterns for school children during holiday weekdays (Mon-Fri), with error bars showing bootstrapped 95% confidence intervals; (B) term weekdays; (C) weekends; (D) adult commuting patterns in same locations as our study, as reported in 2001 UK Census.

During both term time and holidays, the majority of daily movements were under 30 miles (Figs 2A and 2B). However, we saw a significant difference between reported weekday movements in term time and holidays (p < 0.0001 using chi-squared test with 6 degrees of freedom). When compared to holidays, term time movements tended to cluster around short distances; during term time, 76% of daily movements were in the under 1 mile and 1–5 miles categories, compared to 40% in holidays. In holidays, daily movements display a broader distribution, with an increase in both shorter and longer movements (27% of days were spent at home, compared to 5% during term time, and 15% reported distances over 30 miles from home, compared to 3% during term time). Weekend movements appear to be qualitatively more similar to holiday movements than to term time movements (Fig 2C). When comparing our results with commuting patterns reported in the 2001 Census in the electoral wards of our study locations (Fig 2D), we found that adult commuters’ movements resemble school pupils’ movements during term time, with most trips between 1 and 5 miles. However, there were significantly more long distance trips reported by adults in the Census than by children on term weekdays in our study: 16% of reported commuter movements were over 30 miles.

We found that travel movements were different in Easter and half term holidays. During the two-week Easter break, there were more trips of more than 100 miles, and fewer days spent at home (S1 Fig). We also found some differences in travel movements between urban (i.e. based within town or city) and rural schools. Although the overall pattern of movement during term, holidays and weekends was similar, participants in urban areas made fewer long trips during holiday weekdays, and spent fewer days at home at weekends than participants in rural areas (S2 Fig).

**Fig 3:**
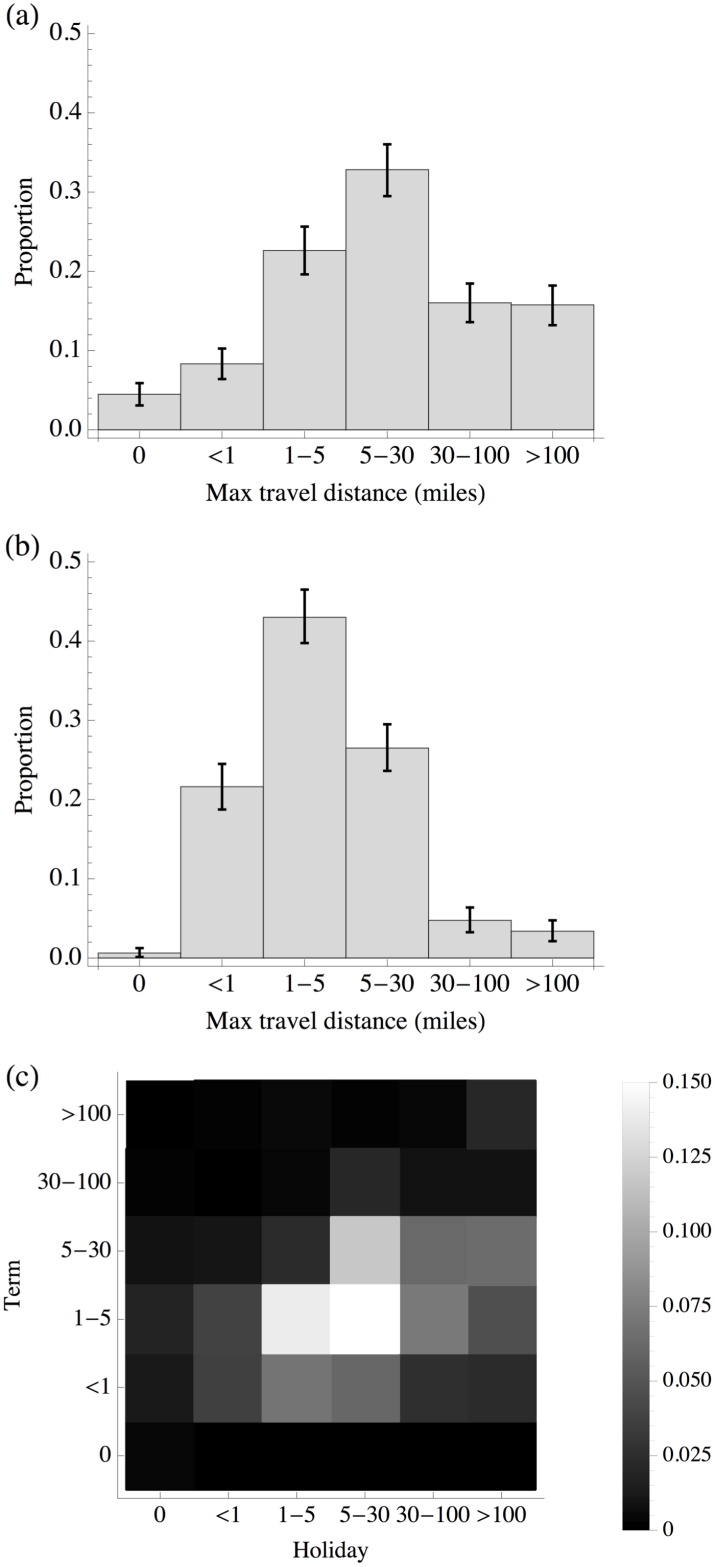
Maximum travel distance reported by each individual over five weekdays (Mon-Fri). (A) Holiday weekdays, error bars show bootstrapped 95% confidence intervals; (B) term weekdays; (C) histogram of maximum distances travelled in term and holiday for each individual in the study. Colour indicates proportion of individuals who travelled each pair of distances.

The maximum distance travelled by an individual during the course of a holiday week was, as might be expected, considerably further than the maximum distance travelled during a term-time week (Figs 3A and 3B). During the holiday week, 31% of participants in the study travelled at least 30 miles, compared with only 8% during the term week. There were also significantly more participants who spent all week at home: 4% did so during the holidays, but less than 1% during term. When comparing the maximum distance travelled during term time and holidays at the individual level (Fig 3C), we see a clear tendency for participants to report travelling further from home during holidays than during term time.

We also examined the accuracy of distance reporting. Participants reported a complete postcode for 3,239 trips, of which 3,092 had matching self reported distances. This made it possible to compare travel distance as measured by postcode with self-reporting for this subset of the data. Each origin and destination was assigned a spatial location as the centroid of the reported postcode area. The Euclidean distance between these centroids was used to calculate a distance category for each trip using the same categories as for self reporting. There was some possible misclassification in all categories. However, overall 85% of trips (2633 reported movements) differed by one or fewer categories from the postcode-estimated distance. The distributions of discrepancies according to distance category are shown in S3 Fig.

## Discussion

This study provides detailed information about the movement patterns of school children in the United Kingdom. We have quantified the range of distances travelled during school term time and school holidays, and shown that holidays are associated with both more days spent at home and with more long distance movements than term time. These patterns are significantly different to those found in the commuting data [20–22] commonly used to parameterise spatial models of human disease transmission. Given the importance of children as drivers of infection [1–5], our results suggest that the geographical spread of disease could in reality differ greatly from existing model predictions.

There are some limitations to our study. Data were self-reported, and it is not clear how accurately participants could identify the distances they travelled. However, assistance was given using relevant local examples when surveys were distributed, and it is likely that participants could make reasonable comparisons between different journeys, which would mean that the trend is reported consistently. The response rate was disappointingly low in some schools, which is perhaps inevitable given the nature of the survey, which had to be completed over the course of a fortnight. We considered the use of web-based data collection, but took the view that this would still require active participation over many days and would potentially exclude participants on access grounds. A survey that could have been answered as a one-off would likely have produced a better response [30], but we would not be confident that activities would have been accurately recalled and, as we have found, there is value in collecting information about movements each day as opposed to—for example—simply asking about the longest movement over the course of a week. Moreover, while movement patterns of school-aged participants appear to be different from reported commuting patterns (Fig 2), weekend and holiday trips made by children are likely to be associated the travel patterns of the parents/guardians. Hence our results suggest that the out-of-term travel patterns of certain adults also differ from the movements reported by commuters in the 2001 Census.

The study was carried out by different student researchers in different schools, and survey methodology may not have been identical in each case. However, many observed differences between schools are likely to be genuine. For example, we found evidence that the movement patterns in large towns were typically more localised than those in rural settings, and that more long distance trips took place in the Easter holiday than in the Spring half term holiday. Therefore it may be inappropriate to model movement patterns by assuming that all holiday periods are identical. However, some effects may have been the result of other unmeasured factors (e.g. local leisure provision or social-demographic factors). Ideally, we would repeat the survey in the same school throughout the year, but this was not feasible within the time constraints of the study.

Despite these limitations, the implications of the results for infectious disease spread are clear. Longer distance movements during holidays offer the potential for infection to move from place to place; such translocations of infection could be facilitated by the movement of either susceptible or infected individuals. Although infected individuals may modify their behaviour [34], the movement patterns described here would apply to pathogens that lead to mild or asymptomatic infections, or those for which individuals are infectious before they are symptomatic.

Our results suggest that term time is associated with intense local spread, whereas holidays, although involving a lower absolute contact rate [2], present opportunities for infection to move long distances. The change in movement patterns is likely to be particularly influential in the case of a school-based outbreak that reaches a high level just before a holiday period, thus releasing a large number of cases to seed infection to new parts of the country. In such an example, school closure—while having the potential benefit of reducing the local force of infection—might have a different effect when considered at a larger spatial scale.

## Supporting Information

S1 Fig Comparison of reported movement distances over half term and Easter holidays. (A) Travel patterns for school children during holiday weekdays (Mon-Fri) over the two-week Easter holiday, with error bars showing bootstrapped 95% confidence intervals; (B) half term weekdays.
(TIFF)

S2 Fig Comparison of reported movement distances in urban and rural settings. (A) Travel patterns for school children during holiday weekdays (Mon-Fri) in urban areas, with error bars showing bootstrapped 95% confidence intervals; (B) urban term weekdays; (C) urban weekends; (D) rural term weekdays; (E) rural weekends; (F) rural holiday weekdays.
(TIFF)

S3 Fig Distribution of the difference between postcode distances and reported distances. Postcode distance is defined as the centroid of the origin postcode area to the centroid of the destination postcode area. Reported distance is the category given from six possible responses in the survey. The difference is the category of the postcode distance minus the reported distance, shown for each of the six distance categories.
(TIFF)

S1 Dataset Reported individual travel patterns. Results from surveys across all schools in the 2011–12 and 2012–13 academic years.
(XLSX)
