# Supplementary figures and images for: School’s Out: Seasonal Variation in the Movement Patterns of School Children
Source: PLoS One. 2015 Jun 1;10(6):e0128070. doi: 10.1371/journal.pone.0128070 (PMC4452697; doi:10.1371/journal.pone.0128070)

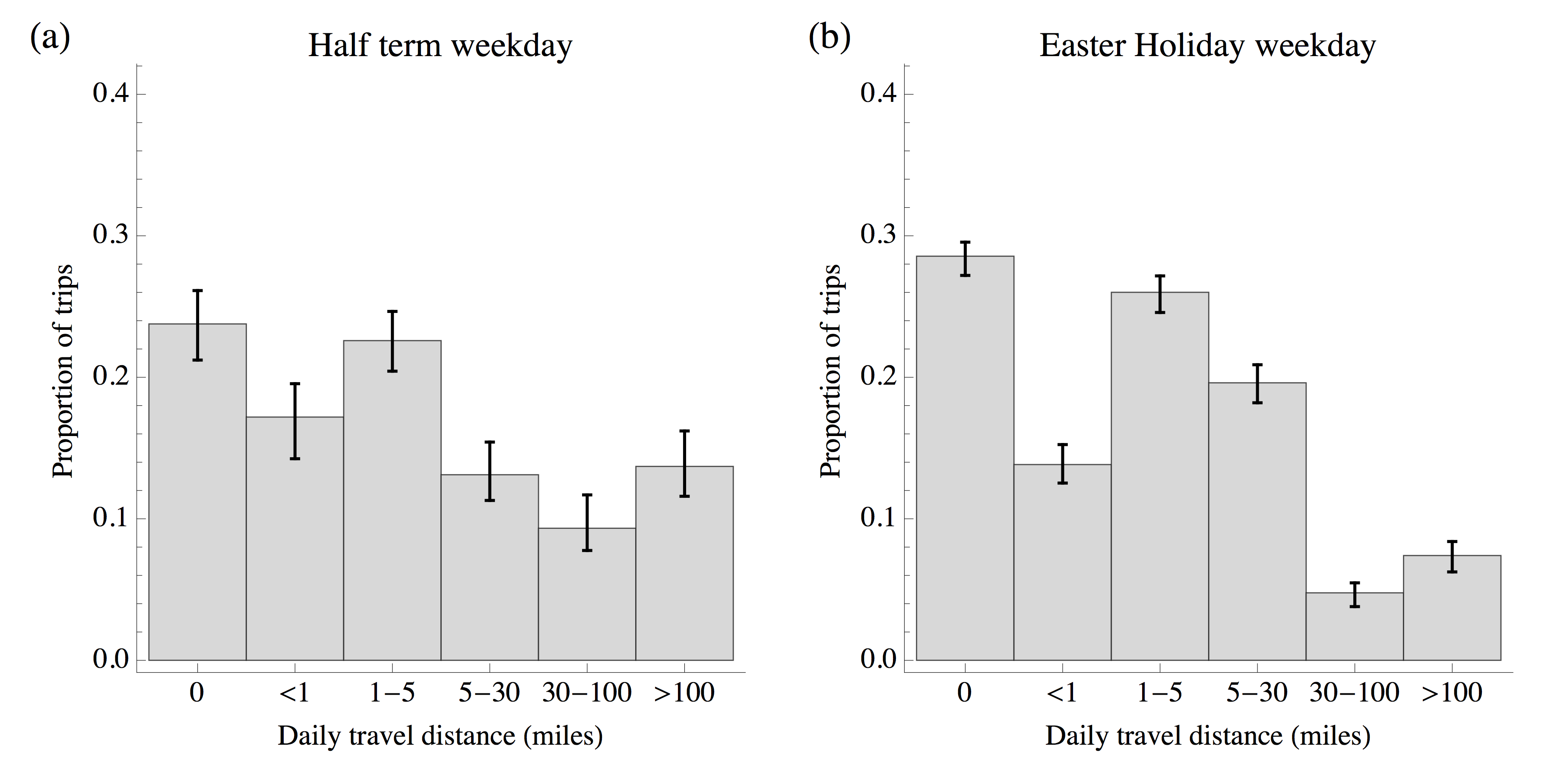

Supplement: S1 Fig — (A) Travel patterns for school children during holiday weekdays (Mon-Fri) over the two-week Easter holiday, with error bars showing bootstrapped 95% confidence intervals; (B) half term weekdays. (TIFF) [file pone.0128070.s001.tiff]

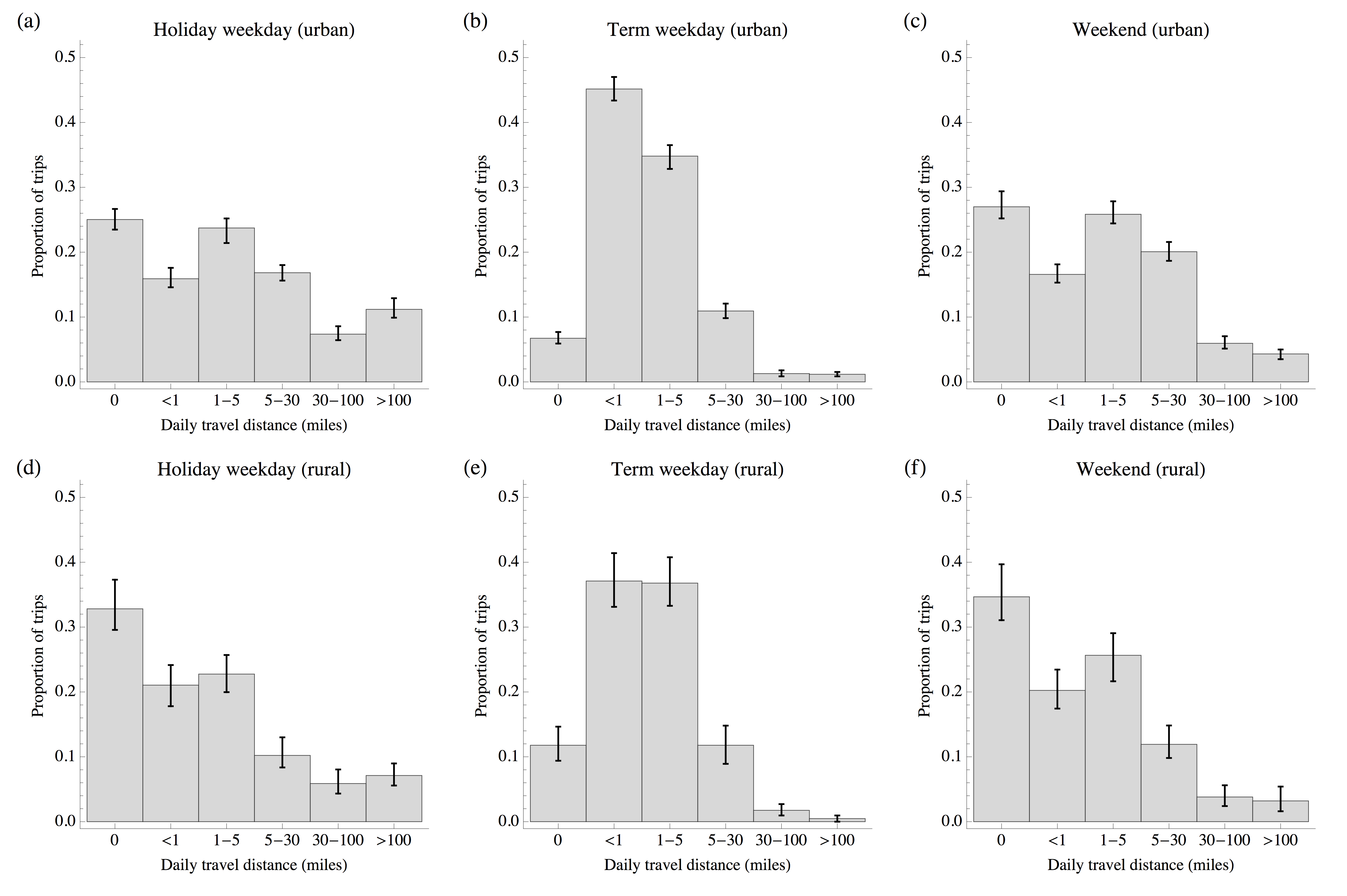

Supplement: S2 Fig — (A) Travel patterns for school children during holiday weekdays (Mon-Fri) in urban areas, with error bars showing bootstrapped 95% confidence intervals; (B) urban term weekdays; (C) urban weekends; (D) rural term weekdays; (E) rural weekends; (F) rural holiday weekdays. (TIFF) [file pone.0128070.s002.tiff]

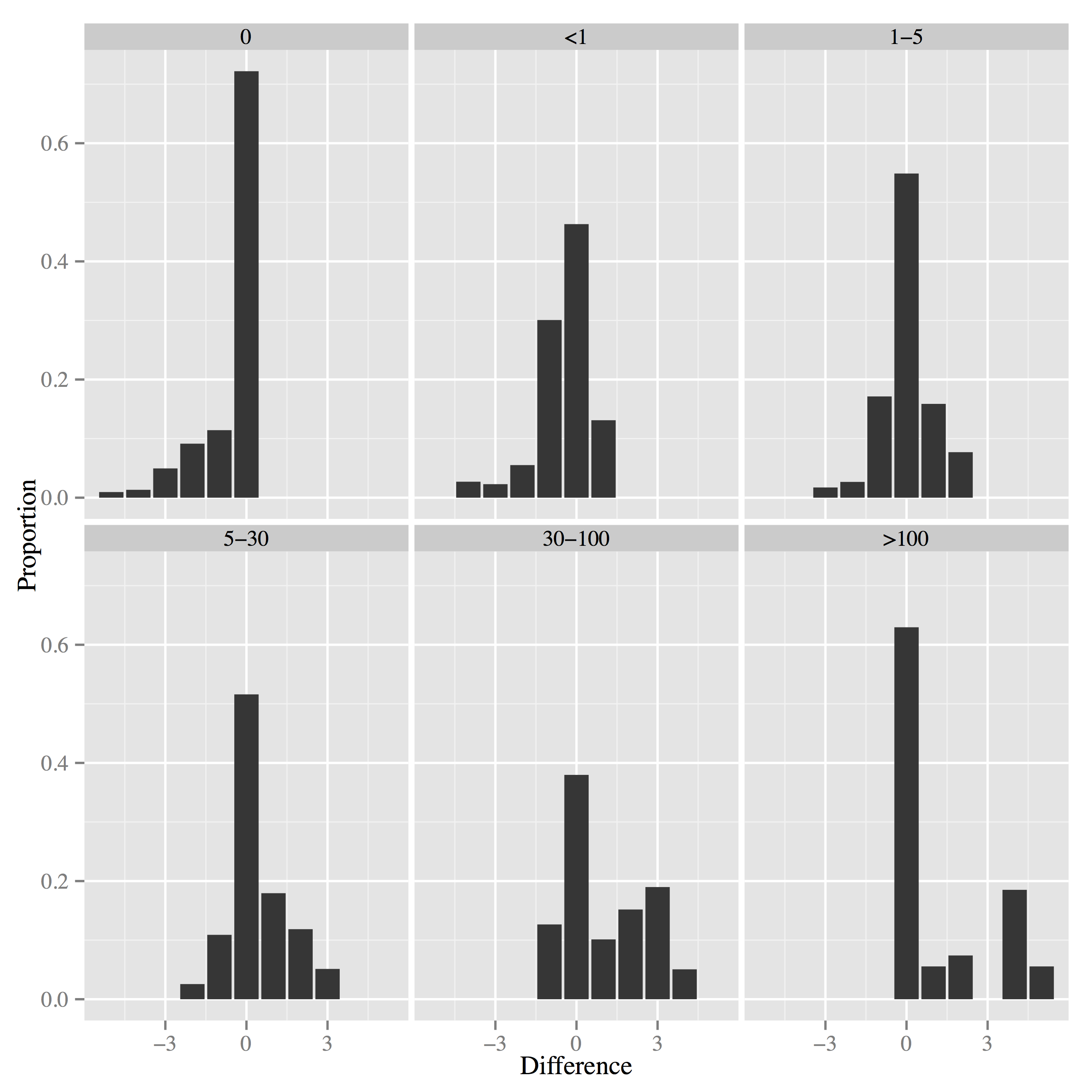

Supplement: S3 Fig — Postcode distance is defined as the centroid of the origin postcode area to the centroid of the destination postcode area. Reported distance is the category given from six possible responses in the survey. The difference is the category of the postcode distance minus the reported distance, shown for each of the six distance categories. (TIFF) [file pone.0128070.s003.tiff]
